# Pyorrhea Alveolaris and Its Treatment

**Published:** 1908-09-15

**Authors:** A. J. MacDonagh

**Affiliations:** Toronto


### PYORRHEA ALVEOLARIS AND ITS TREATMENT.

BY A. J. MACDONAGH, D.D.S., TORONTO.

After having observed as closely as possible for some years, I have come to the conclusion that there are two or three different kinds of diseases coming from many different causes, called pyorrhœa.

We have the condition which exists when the pericementum dies and the alveolus dissolves or is absorbed away without much deposit, if any, of a lime salt nature, having been left on the root. This condition very often follows a severe nervous strain, such as business worry, nervous prostration, great anxiety or grief. It also follows constipation, that is, chronic constipation, and the reason it follows in the wake of these disorders is plain when we consider the anatomy of the parts implicated.

The pericemental tissue is confined in a very small space between the hard tooth substance and the hard bony subtance of the alveolus. In normal conditions the blood has ample room to flow in and out without congestion. There is very little flow into the tooth substance, however, as the pulp supplies all the nourishment the tooth requires when the pulp is alive. That condition of things is changed the moment you change the beat of the heart. The moment the heart starts to pump, blood is forced into this tissue, the vessels are engorged and hyperaemia of the pericementum is the logical consequence. As long as the pericementum is normally filled with blood, micro-organisms cannot attack it, but just as soon as it is anaemic or hyperaemic, bacteria which are always in contact with it can get a foothold and start a little colony producing their ptomaines and toxins which poison the pericementum and cause a further breaking down in which further habitations for the bacteria are possible, and so on until the whole pericementum is destroyed.

During this paper we will use the word "Pyorrhœa", because at the present time there is no other name which will designate the disease which we know as pyorrhœa. When we consider that this form of pyorrhœa (so-called) is probably more prevalent than any other form, it immediately leads us to contemplate the writings of men who claim that pyorrhœa is due entirely to lime salts having ingress at the gingival margin, or that pyorrhœa is always the result of uratic deposit from the blood.

That there is a deposit from the blood, a deposit of lime salts after the blood has oozed into the injured part and broken up, we need not have the slightest doubt. That kind of deposit is often there in the condition of pyorrhœa just described, particularly so if that condition has existed for any length of time, say a year or more. It is also quite easy to see how lime salts could enter such a cavity from the saliva and form deposits on the root.

But to assert that the deposit is always there in large enough quantities to cause the disturbance seems to me, with the experience I have had in the treatment of pyorrhœa, to be going a long distance out of our way to find a local cause. When I was here last December I brought accurate records of 642 cases which I had treated in the year and four months previous—nearly 80 per cent, of which had little or no deposits on the root.

To cure this condition of pyorrhœa, it has been claimed that I use my scalers and scrapers to remove the deposit and that therefore I am removing the cause so that the disease will disappear. But when we consider the conditions for a moment we will readily understand that although there is not a particle of lime salts on the roots, nevertheless, on account of the dead pericementum and the millions of micro-organisms and their habitations, if we desire any sort of success, let alone a permanent cure, we must most assiduously apply ourselves to the removal of all dead, decaying and foreign material. Not only that, but in the majority of cases, we must currett the living tissue or cauterize where it is impossible to currett, in order that we get new and healthy granulations.

I know that in advocating the origin of this disease in bacterial action on anaemic or hyperaemic pericementum, made possible by the opsonic index being lowered, I am combatting the idea of some of the best and greatest investigators in the subject and of course, may be wrong, but I am depending not so much on my knowledge of anatomy or urinary analysis as upon the records of my cases and my power of observation. If the statistics I have kept show anything, they most certainly indicate that the line of argument I have just used is the correct one. Dr. Talbot speaks of the alveolus being an end tissue, and that it is through faulty elimination as affecting the blood supply, resorbed and dissolved away, but he does not take into consideration the fact that the place where we find pyorrhœa is usually in the pericementum, and we have often seen, many of us, the alveolus standing partially in place and a pocket existing between it and the root of the tooth. You are often able to run your instrument up there and turn it toward the buccal or lingual side and find a solid part of the alveolus still in place and doing good service as far as it can.

That the alveolus is sometimes prematurely resorbed, I most certainly believe. I have in my own practice, and probably some of you have in yours, cases where this fact is demonstrated, but in these cases—and they are found usually in athletes who have done a great amount of training—in these cases, I say, the teeth stand in position, the pericementum is intact, but the whole structure is loose and the teeth move with the pericementum and gum tissue. There is no discharge at the roots, and the gums are attached to the teeth. I had a case sent to me by one of our Toronto dentists during the last year, of one of our champion golf players who was affected in this way. I have had three golf players affected in the same way, and yet golf is supposed to be a very healthy game.

Of course, in an over-trained athlete, particularly one of nervous disposition, we are likely to have pyorrhœa in the ordinary form.

Just here I want to say that it is not my desire to belittle the work of Dr. Talbot, or Dr. D. D. Smith, or Dr. Younger, nor any of those who have given time and energy to clearing up the subject, each one I believe has discovered certain truths concerning the origin of the disease, which makes it easier for other investigators to follow in their line.

A form of this disease which we sometimes find is no doubt due to rheumatic or gouty diathesis. In that form calcic deposit is formed on the root between the gingival margin and the apex, not having an opening at the gingival margin. In fact, it is very often mistaken for alveolar abscess, but upon testing the tooth you find the pulp is alive, and you have to make an opening through the gum over the affected part to succeed with the case. This is a very annoying form and is apt to cause serious constitutional disorders, if extensive, on account of the absorption which is likely to take place.

I had one patient, a gentleman who informed me that a bad attack of pyorrhœa always followed an attack of rheumatism, and after his pyorrhœa was cured the rheumatism disappeared, and this was without changing his diet or taking other constitutional treatment.

I have had a number of patients who were cured of rheumatism after being treated for pyorrhœa, but they were also treated constitutionally for the rheumatism.

Another form of pyorrhœa which we find is due to brushing the teeth improperly, or to the use the of silk floss when not properly understood, also to ligaturing and to separating the teeth. Malocclusion is also a prolific cause, and of course when the teeth are hard and dense and the pericementum thin and anaemic. All of these causes are of course local.

There has been an idea that the pericementum was about the same thickness around all teeth. I have a friend in

Toronto who does nothing but extract teeth. He has been supplying me with teeth freshly extracted, and we have been together looking into the thickness of the pericementum, and there is a wonderful difference in the pericementum of patients about the same age.

If patients have too hard a brush and drive underneath the gum, the materials which they should wash off the teeth and out of the mouth, namely, food debris and micro-organisms, you are very likely to have sufficient irritation in the pericementum if conditions are at all favorable to start the trouble. And just at this point, gentlemen, it is well to say a word about brushing the teeth, as it is one of the great factors in keeping teeth healthy which have been affected by pyorrhœa.

On account of my radical opposition to the usual method of brushing the teeth, which I have held for the last ten or fifteen years, it has been said I did not believe in brushing the teeth, and at many conventions I have had to combat that idea. However, at the present time there are many men taking the stand I do, and I am not now accused of the heresy. The brushes which are used are nearly all too large. You cannot manipulate them properly in your mouth, and in order to preserve the teeth as well as the gums you must brush always from the gums onto the teeth, and you must do it thoroughly. In order to clean the lower anterior teeth at the distal gingival margin you must have a brush with not more than 4 rows square bristles of the ordinary tooth brush, but if that brush is used faithfully, after every meal and before retiring, the patients' teeth will never accomulate tartar in that position. A great many of my patients are faithful in this and have no tartar on the anterior teeth at all, and they tell me it is no great trouble to brush the teeth in the proper manner. They used to have a great deal of trouble before and they are, of course, very thankful for the information.

There are two methods of treatment which I believe are successful. One is called the Opsonic Method and the other the Local. Of course we hear of constitutional treatment outside the Opsonic treatment through advertisements of patent medicines, but in practice we pay no attention to them. The Opsonic method I will not speak of tonight, I want to speak of the method I use.

In the first place, we have to consider what method to use to reduce the sensitiveness and minimize the pain in operating. A few years ago the pain was considered so great that it was an easier matter to extract the teeth than have them treated. However, in our operations at the present time we cause very little pain, partly by using good instruments, and principally by knowing how. Sometimes we use an anæsthetic called Nervocidin, a drug obtained from a plant in South Africa called the Gasu-Basu plant. This drug has only come into use in recent years and is practically only of use to dentists. A great deal has been written about it by men who used it and I fear by men who never had any experience with it. It is a dangerous drug if not handled carefully, and intelligently. I have seen quite a large tract of bone destroyed by its evil influence, and have seen the pulp in a tooth destroyed when such destruction was not desired. It is given us as a harmless anæsthetic, but when such results follow its use, and when we know it is highly toxic, its harmlessness must be taken comparatively. It is a very powerful anæsthetic when applied topically either to tooth substance or to gum or bony tissue and as such it is a good servant, but we must always remember that if placed in a deep cavity in a tooth, that we are likely to have the pulp devitalized as well as anæsthetized. To use it in pyorrhœa we make a solution of about 1 in 500 to 1 in 1,000 and after winding some cotton around a broach, dip it in the solution and place it rapidly in the bottom of the pocket. Then go ahead with your instruments and scale off any deposits which may require scaling and which will not cause much pain, allowing your anæsthetic ten minutes or more to do its work, of course being careful that none of it is mixed with the saliva or allowed to escape into the mouth, lest you have the muscles of the throat paralyzed and a miserable, suffocating feeling overcome your patient, which will last for several hours. I had one patient four hours in that suffocating condition and if I had used more probably I should have had a Coroner's Jury to face. One feature about this anæsthetic is that the anæsthesia does not pass away quickly.

Taking it for granted that the instruments are thoroughly sterilized, and as a method of sterilization we prefer boiling the instruments in a receptacle made for the purpose which is in plain view of the patient, just the same as we believe in thoroughly washing our hands and of not being afraid the patient will know it.

In the great majority of teeth the most sensitive part of the tooth is that part just at the gum margin where the enamel ceases to exist and the cementum starts. The cementum, of course, should be covered by the gum tissue, but we find it very often not covered and hyper-sensitive. The enamel also at that junction is sensitive, exceedingly so, and our great care in using our instruments must be not to pass over this area any oftener than it is impossible to avoid, but while working in the pockets, keep the instruments as far down as necessary. This necessitates a rotary motion, which after a time becomes easy to you. I say rotary motion—I cannot describe it very well. The whole object is to work the instrument on the root laterally and keep from moving your instrument up over that sensitive part—keep from going past the gingival margin any oftener than necessary.

I have been asked to describe the instruments I use. There is nothing new about them particularly, except my files. I may have changed the shapes a little from the originals and have numbered them to suit my own convenience, but, as a matter of fact, they are with some slight changes copied from Tompkins' and Youngers' instruments and can be obtained at almost any dental depot. I have them with me and will be pleased to show them to you.

I do not think it matters so much the instruments you use as the use of the instruments. Dr. Hutchinson has some which are very fine and there are a number of others on the market which will answer the purpose as well, but I have grown accustomed to the ones I have and use them to the best advantage.

At first I used Tompkins' a great deal, and then Younger's, then I used Mill's prophylactic instrument, and I still use that on account of the rapidity of its motion in certain cases, but it is not good for the finer work I have to do, as a rule, so it is not used often in my practice.

The first step of course is to remove all deposits which are attached to the tooth, also all the dead pericementum, and we have used our instruments to detach any particle of dead bone which might be lying around. Sometimes the dead bone is not attached to the tooth. Very frequently I find little pieces of dead bone attached to what we might call the outside of the pocket.

The next step is to be sure we have two freshened surfaces, if we want them to unite, therefore it behooves us to consider the surfaces we have before us. We find that the mouths of the canaliculi of the roots have become closed by lime salts, whether from the blood or the saliva it matters not, and the small tendrils of animal matter in the canaliculi are sealed in. Your scraping, of course, if well done, has to a degree removed these seals, but it is practically impossible, under the circumstances, to have done it entirely. You can find that out by taking a tooth which has been affected with pyorrhœa, put it in a little rubber tube— around the root—take your instrument and scrape that root as thoroughly as you can, take off your tube and see how well you have succeeded.

You find also that the gum tissue is not in a very healthy condition, nor the bony tissue. In fact, the walls of your pus pockets or the parts of them which remain are laden with micro-organisms and their by-products, all of which must be removed, and also if you have used Nervocidin, you have a dangerous drug lying there which, of course, you must make provision to get rid of.

To dissolve the lime salts we use either Hydrochloric, 75 per cent, or Sulphuric Acid, or both, and to remove the objectionable animal matter and make a fresh scar use Tri-chloracetic, saturated, full strength. In using these drugs of course you must be careful; they are very powerful. There is one little point, not essential to the treatment, but which I have found of value: If you use hydrochloric acid on the tooth without any precaution, after the operation is over the tooth will be exceedingly sensitive to heat and cold and to the touch, almost impossible to brush it. In order to overcome that I dry the tooth as thoroughly as I can and then varnish it before using the acids. Harvardid Varnish is a fine thing for that purpose, but it is a little difficult for us to get it. You can use shellac. The varnish will run over on the gum, take your compressed air or blow syringe and harden the shellac, then take a little instrument and cut away the shellac which is just overlapping the gum, because if you do not it will be an additional irritant. You have now protected the part of the tooth you desire protected.

I was asked to-night if I ever found an attachment between the teeth which were treated and the gum tissue after it had been treated. I would say yes, I do. In the great majority of cases there is an attachment. I do not say the periosteum or pericementum grows again. In order to decide that point it would be necessary to resort to dissection, and as none of my patients whom I could use for that purpose have died, so far as I know, I have not been able to do so.

Patients who have exhibited themselves for me at our conventions in Toronto have proven, however, that you can not run an instrument between tooth and gum tissue without drawing blood and as far as you can see in that sort of examination the attachment is a direct one.

Dr. Bogue told me to-night he had seen attachment in two of his cases, I was glad to hear it. I had not met any person before who had used his method and obtained an attachment. The hydrochloric acid I use is 75 per cent. I would use it pure only for the inconvenience that if there is only a trace of ammonia in the breath you will have disagreeable fumes, and if inhaled they are very irritating to the bronchial tubes, whereas if you put enough water with it at the start, you do not get those fumes.

Teach your patient how to brush his teeth and be sure you impress it on him. Make him feel how necessary it is. Have him understand that if any food material is allowed to get between the gum and the tooth tissue there will be no healing. I take the brush myself and stand beside my wash basin and show my patient how to brush the teeth. I brush my own teeth and the next time he comes back, if his teeth are not thoroughly clean—well, sometimes I make enemies, I say things in a strong manner, and if he comes back the second time and has not done what I want, I refuse to work for him. He either must do what I say while he is my patient or he becomes someone else's patient. They have gone to other dentists, who saw what was the trouble, and sent them back to me, so they were between the devil and the deep sea, and they chose the devil—which was me.

In cases where the patient is inclined to be anæmic we must resort to massage. Find out, if possible, the cause of the pyorrhœa, make our recommendations accordingly, and warn patient against bad habits.

If necessary advise your patient to see his physician. A dentist in taking care of a patient will often be able to forewarn him. Sometimes we are rebuffed, but generally it is all right.

A colonel in one of our regiments came to me for treatment. I saw his gums were not responding properly to the treatment and I sent him to his physician for examination.

I told him to have the urine properly analyzed and he would know his condition. His physician told him there was nothing wrong, to pay no attention to what I said.

In two weeks' time the gentleman was in the hospital. He stayed there eight weeks and was in very bad shape. Since that time the physician has been telling everybody in town that the dentists know more than he thought they did. I do not suppose you have that trouble here, but we do over there—that we do not always get recognition from the physician. Perhaps it is good for their business.

Often during my practice I have patients sent to me whose teeth are apparently begond redemption, so much of the bone and peridental membrane and in fact the periosteum are gone, that to expect a rebuilding would be folly. However, there are some teeth comparatively solid and we desire to retain and make useful those which are exceedingly loose, It is quite possible to do so in many cases, particularly is it feasible if, say, the two or four upper or lower incisors are the shaky ones.

What we must do is to resort to replantation, but replantation, if not well understood, is often a failure. I have been gratified lately to have dentists tell me that they have seen teeth I replanted years ago doing good service to-day.

I may say I have been replanting teeth for nearly twenty years and so far as I know have had very few roots resorbed, and in every case where resorption has occurred, it has been my fault. However, where teeth are affected by pyorrhœa to such a great extent, if we replanted them in the ordinary way, expecting them to remain of their own attachment to the jaw, the operation would be a failure. What we must do in those cases is to treat the roots of loosened teeth, when extracted, as we would do a porcelain root, that is, make them perfectly smooth, remove all peridental tissue and having thoroughly and completely filled the root, attach them to a bar which extends into the pulp chamber and which is soldered to a gold plate sufficiently strong to withstand the strain of mastication and which is fastened permanently to the solid teeth. Make your attachment in the solid teeth and attach the loose teeth to it by running the bar on the lingual side, showing no gold, of course.

A word regarding the technique of the operation. After extracting the teeth we drop them into 4 per cent formaldehyde for four minutes or so, take our instruments and scrape the root clean. Then if the tooth had elongated, cut enough off the apical end to allow for shortening the tooth the distance it was elongated, and having fitted our splint to see that everything is all right, take an instrument, which we have run through the flame to sterilize, go down into the tooth socket, break up the blood clot that is there and start new blood flowing. At the same time or as soon as possible, dip the root into hydrochloric acid 75 per cent pure, have your assistant nearby, and cement mixed ready. Fill the cavities which are to take the retaining appliance with cement, press the retaining appliance home, using a small pair of plyers to bring your implanted teeth into alignment,

If you do this work faithfully and well, you will have the teeth in place, and they will be much superior to any bridge from the patient's standpoint. You will not have resorption, as the roots will be only touching gum tissue. You will not have shrinkage of the gums because they will cling around the root of the tooth as if they had grown to it, and in some of my cases they have certainly grown to the root, although it is not absolutely necessary.—*The Journal.*

